# Stage-dependent differential gene expression profiles of cranial neural crest-like cells derived from mouse-induced pluripotent stem cells

**DOI:** 10.1007/s00795-019-00229-2

**Published:** 2019-07-11

**Authors:** Ayano Odashima, Shoko Onodera, Akiko Saito, Yuuki Ogihara, Tatsuya Ichinohe, Toshifumi Azuma

**Affiliations:** 1 Department of Oral Health Science Center, Tokyo Dental College, 2-9-18 Misaki-cho, Chiyoda-ku, Tokyo, 101-0051 Japan; 2 Department of Biochemistry, Tokyo Dental College, Tokyo, Japan; 3 Department of Dental Anesthesiology, Tokyo Dental College, Tokyo, Japan; 4 Department of Biochemistry, 2-9-18 Misaki-cho, Chiyoda-ku, Tokyo, 101-0051 Japan

**Keywords:** Cranial neural crest, Migratory neural crest, iPS cells, RNA sequencing, Adamts

## Abstract

Cranial neural crest cells are multipotent cells that migrate into the pharyngeal arches of the vertebrate embryo and differentiate into various craniofacial organ derivatives. Therefore, migrating cranial neural crest cells are considered one of the most attractive candidate cell sources in regenerative medicine. We generated cranial neural crest like cell (cNCCs) using mouse-induced pluripotent stem cells cultured in neural crest-inducing medium for 14 days. Subsequently, we conducted RNA sequencing experiments to analyze gene expression profiles of cNCCs at different time points after induction. cNCCs expressed several neural crest specifier genes; however, some previously reported specifier genes such as paired box 3 and Forkhead box D3, which are essential for embryonic neural crest development, were not expressed. Moreover, ETS proto-oncogene 1, transcription factor and sex-determining region Y-box 10 were only expressed after 14 days of induction. Finally, cNCCs expressed multiple protocadherins and a disintegrin and metalloproteinase with thrombospondin motifs enzymes, which may be crucial for their migration.

**Electronic supplementary material:**

The online version of this article (10.1007/s00795-019-00229-2) contains supplementary material, which is available to authorized users.

## Introduction

Stem cell-based tissue engineering is important in the field of oral science because it facilitates the regeneration of damaged tissues or organs [1, 2]. Various stem cell populations exhibiting regeneration potential in the craniofacial region have been identified. Of these, cranial neural crest cells (cNCCs) are considered one of the most important candidates owing to their role in craniofacial tissue organization [3]. cNCCs comprise a multipotent population of migratory cells that are unique to the vertebrate embryo and can differentiate into various craniofacial organ derivatives [4, 5]. The neural crest (NC) can form teratoma when transplanted into immunocompromised animals [6]. cNCC development involves three stages [7–10]: the neural plate border stage, the premigratory stage, and the migratory stage. During the migratory stage, cNCCs delaminate from the posterior midbrain and individual rhombomeres in the hindbrain [11] and migrate into the pharyngeal arches to form skeletal elements of the face and teeth and contribute to formation of the pharyngeal glands (the thymus, thyroid, and parathyroid) [12]. Therefore, cNCCs presumably represent a new treatment strategy for diseases of the craniofacial region [13].

Development from the premigratory to migratory stage proceeds swiftly [14]; thus, it is typically difficult to detect the precise time point of this transition [15]. A recent transcriptome analysis of pure populations of migratory cNCCs cells expressing the sex-determining region Y-box 10 (*Sox10*) from chicks [16] has substantially improved our understanding of cNCC characteristics. However, whether these cells are in the migratory stage and how long it takes to promote embryonic stem (ES) cell-derived NCCs from the premigratory to migratory stage remains unclear. In recent years, the use of induced pluripotent stem (iPS) cells as a revolutionary approach to treat various medical conditions has garnered much attention [17, 18], and iPS cells as a cell source have shown several evident advantages over ES cells and primary cultured cNCCs in regenerative medicine [16]. In addition, embryonic NC development depends on several environmental factors that influence the regulation of NC progenitors and timing of differentiation; therefore, it is important to elucidate the regulatory gene networks and expression profiles of mouse iPS (miPS) cell-derived cNCCs. Recent advances in next-generation RNA sequencing (RNA-seq) technologies have facilitated comprehensive analysis of gene expression profiles [19–21]. Therefore, in the present study, we used RNA-seq to investigate the gene expression landscape of cNCCs induced from miPS cells. We treated iPS-derived cells with cNCC induction medium for 14 days and performed RNA-seq experiments. Our results indicated that *c*-*Myc*; ETS proto-oncogene 1, transcription factor *(Ets1)*; *Sox10*; a disintegrin and metalloproteinase domain metallopeptidase with thrombospondin motifs (*Adamts*) 2 and 8; protocadherin alpha (*Pcdha*) 2, *5*, -7, -11, and -12; protocadherin alpha subfamily C,1 (*Pcdhac1*); and protocadherin gamma subfamily C,3 (*Pcdhgc3*) may be appropriate markers for migratory cNCCs induced from miPS cells.

## Materials and methods

### miPS cell culture

The miPS cells used in the present study (APS0001; iPS-MEF-Ng-20D-17 mouse-induced pluripotent stem cell line) were purchased from RIKEN BRC (Ibaraki, Japan) [22]. The cells were incubated with inactivated murine embryonic fibroblast (MEF) feeder cells in Dulbecco’s Modified Eagle’s Medium (DMEM; Invitrogen, Carlsbad, CA, USA) supplemented with 15% KnockOut™ Serum Replacement (Invitrogen), 1% nonessential amino acids (Chemicon, Temecula, CA, USA), 1% l-glutamine (Chemicon), 1000 U/mL penicillin–streptomycin (P/S; Invitrogen), and 0.11 mM 2-mercaptoethanol (Wako Pure Chemical Industries Ltd., Osaka, Japan); 60-mm cell culture plates were used for passaging the cells at a density of 1 × 10^5^ cells/plate. Cells were grown in 5% CO_2_ at 95% humidity, and the culture medium was changed each day.

### Embryoid body (EB) formation and cNCC differentiation

**Fig. 1:**
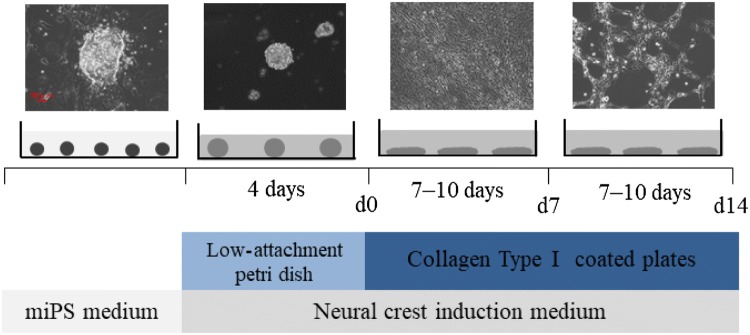
The experimental protocol used to induce the formation of cranial neural crest cells (cNCCs) from mouse-induced pluripotent stem (miPS) cells. The photographs show miPS cells at four different stages: initial miPS cells, embryoid body (EB) on day 0 (d0), and cNCCs on d7 and d14. Small circles represent miPS cells; large circles represent EBs; ellipses represent d7 and d14 cells. Scale bar 50 μm

We obtained cultured cNCC cells as described previously [23] (Fig. 1). miPS cells were dissociated with 0.05% trypsin–ethylenediaminetetraacetic acid (EDTA; Invitrogen) and transferred to low-attachment, 10-mm Petri dishes at a density of 2 × 10^6^ cells/plate to generate EBs. The generated EBs were cultured in cNCC induction medium comprising a 1:1 mixture of DMEM and F12 nutrient mixture (Invitrogen) and then in Neurobasal™ medium (Invitrogen) supplemented with 0.5 × N2 (Invitrogen), 0.5 × B27 (Invitrogen), 20 ng/mL basic fibroblast growth factor (Reprocell, Yokohama, Japan), 20 ng/mL epidermal growth factor (Peprotech, Offenbach, Germany), and 1% P/S for 4 days; the medium was changed every other day. After 4 days, day 0 (d0) EBs were collected and transferred to 60-mm cell culture plates coated with 1 μg/mL collagen type I (Advanced BioMatrix, San Diego, CA, USA). The cells were then subcultured in the same medium; the medium changed every other day, and any rosette-forming cells were eliminated. After 7–10 days, d7 cells were dissociated with 0.05% trypsin–EDTA and transferred to 60-mm cell culture plates coated with 1 μg/mL collagen type I at a density of 1 × 10^5^ cells/plate to generate d14 cells. This process was repeated three times. The cells from each of these passages were collected for RNA extraction.

### O9-1 cell culture

O9-1 cells, a mouse cNCC line, were purchased from Millipore (Billerica, MA, USA) and cultured as a control, as previously described [24].

### RNA extraction and quantitative reverse transcription polymerase chain reaction analysis (qRT-PCR)

The expression of representative NC markers, namely nerve growth factor receptor (*Ngfr*), snail family transcriptional repressor (*Snai*) 1 and 2, and *Sox9* and *10*, was analyzed using qRT-PCR analysis. Total RNA was extracted using QIAzol^®^ reagent (Qiagen, Valencia, CA, USA) according to the manufacturer’s protocol, and RNA purity was assessed using NanoDrop^®^ ND-1000 spectrophotometer (Thermo Fisher Scientific, Waltham, MA, USA). Each RNA sample exhibited an A260/A280 ratio of > 1.9. Complementary DNA (cDNA) was synthesized using a high-capacity cDNA reverse transcription kit (Applied Biosystems, Foster City, CA, USA), and qRT-PCR analysis was performed with Premix Ex Taq™ reagent (Takara Bio Inc., Otsu, Japan) according to the manufacturer’s protocol using Applied Biosystems^®^ 7500 Fast Real-Time PCR System; the primer sequences are presented in Table 1. All samples were normalized to 18S ribosomal RNA levels. Relative expressions of genes of interest were analyzed using the ΔΔ*C*t method and were compared among the groups using analysis of variance, followed by the Bonferroni test when significant differences were detected among the groups. A significance level of *p* < 0.05 was used for all analyses, and all data were expressed as mean values and standard deviations.

**Table 1:** Primers used for quantitative reverse transcription polymerase chain reaction (qRT-PCR)

Gene	Forward primer sequence	Reverse primer sequence
*18S rRNA*	CGGACAGGATTGACAGATTG	CGCTCCACCAACTAAGAACG
*Ngfr* (*p75NTR*)	ACTGAGCGCCAGTTACGC	CGTAGACCTTGTGATCCATCG
*Snail* (*Snail*)	CTTGTGTCTGCACGACCTGT	AGGAGAATGGCTTCTCACCA
*Snai2* (*Slug*)	CATTGCCTTGTCTGCAAG	CAGTGAGGGCAAGAGAAAGG
*Sox9*	GTACCCGCATCTGCACAAC	CTCCTCCACGAAGGGTCTCT
*Sox10*	ATGTCAGATGGGAACCCAGA	GTCTTTGGGGTGGTTGGAG

### Immunohistochemistry

The cells were fixed with 4% paraformaldehyde (Wako Pure Chemical Industries Ltd.) for 15 min followed by methanol (Wako Pure Chemical Industries Ltd) for 5 min. After washing, the nonspecific binding of antibodies was blocked by adding 5% bovine serum albumin (BSA; Wako Pure Chemical Industries Ltd.) in a phosphate-buffered saline with 0.5% Triton X-100 (PBST) for 1 h. The cells were then incubated with the primary antibodies Snai1 1:50 for Rabbit polyclonal anti-Snai1 (26183-1-AP; Proteintech Group, Inc. Chicago, IL, USA.) and Sox10 1:500 for Mouse monoclonal anti-Sox10 (AMAb91297; Atlas Antibodies, Bromma, Sweden.) in PBST for 2 nights at 4 °C. We conducted that the positive control of Snai1 was O9-1 cells (cranial neural crest cells) and the positive control of Sox10 was DP cells (dental pulp cells). The negative control of Snai1 and Sox10 was SNL cells (fetus fibroblast cells) (Fig. S1).They were then incubated in the secondary antibodies fluorescein isothiocyanate Alexa Flour 488-conjugated affinity purified Goat anti-Rabbit IgG (H&L) (ab150077; Abcam, Cambridge, MA, USA) at a dilution of 1:500 for Snai1 and Alexa Flour 568-conjugated affinity purified Goat anti-Mouse IgG (H&L) (A-11004; Invitrogen) at a dilution of 1:500 for Sox10 in PBST for 1 h. Eventually, the cells were stained with 4,6-diamidino-2-phenylindole (DAPI; Sigma, Livonia, MI, USA) to visualize the nuclear DNA.

### RNA-seq

Total RNA from each sample was used to construct libraries with the Illumina TruSeq Stranded mRNA LT Sample Prep Kit (Illumina, San Diego, CA, USA), according to the manufacturer’s instructions. Polyadenylated mRNAs are commonly extracted using oligo-dT beads, following which the RNA is often fragmented to generate reads that cover the entire length of the transcripts. The standard Illumina approach relies on randomly primed double-stranded cDNA synthesis, followed by end-repair, dsDNA adapter ligation, and PCR amplification. The multiplexed libraries were sequenced as 125-bp paired-end reads using the Illumina Hiseq 2500 system (Illumina). Prior to performing any analysis, quality of the data was confirmed and read cleaning, such as adapter removal and simple quality filtering, was performed using Trimmomatic (ver. 0.32). Subsequently, the paired-end reads were mapped to the mouse genome reference sequence GRCm38 using the Burrows–Wheeler Aligner (ver. 0.7.10). The number of sequence reads mapped to each gene domain using SAM tools (ver. 0.1.19) was counted, and the reads per kilobase of transcript per 1 million mapped reads (RPKM) for known transcripts were calculated to normalize the expression level data to gene length and library size, thereby facilitating the comparison of different samples.

## Results

### Gene expression profiles and immunohistochemistry of cNCCs derived from miPS cells

**Fig. 2:**
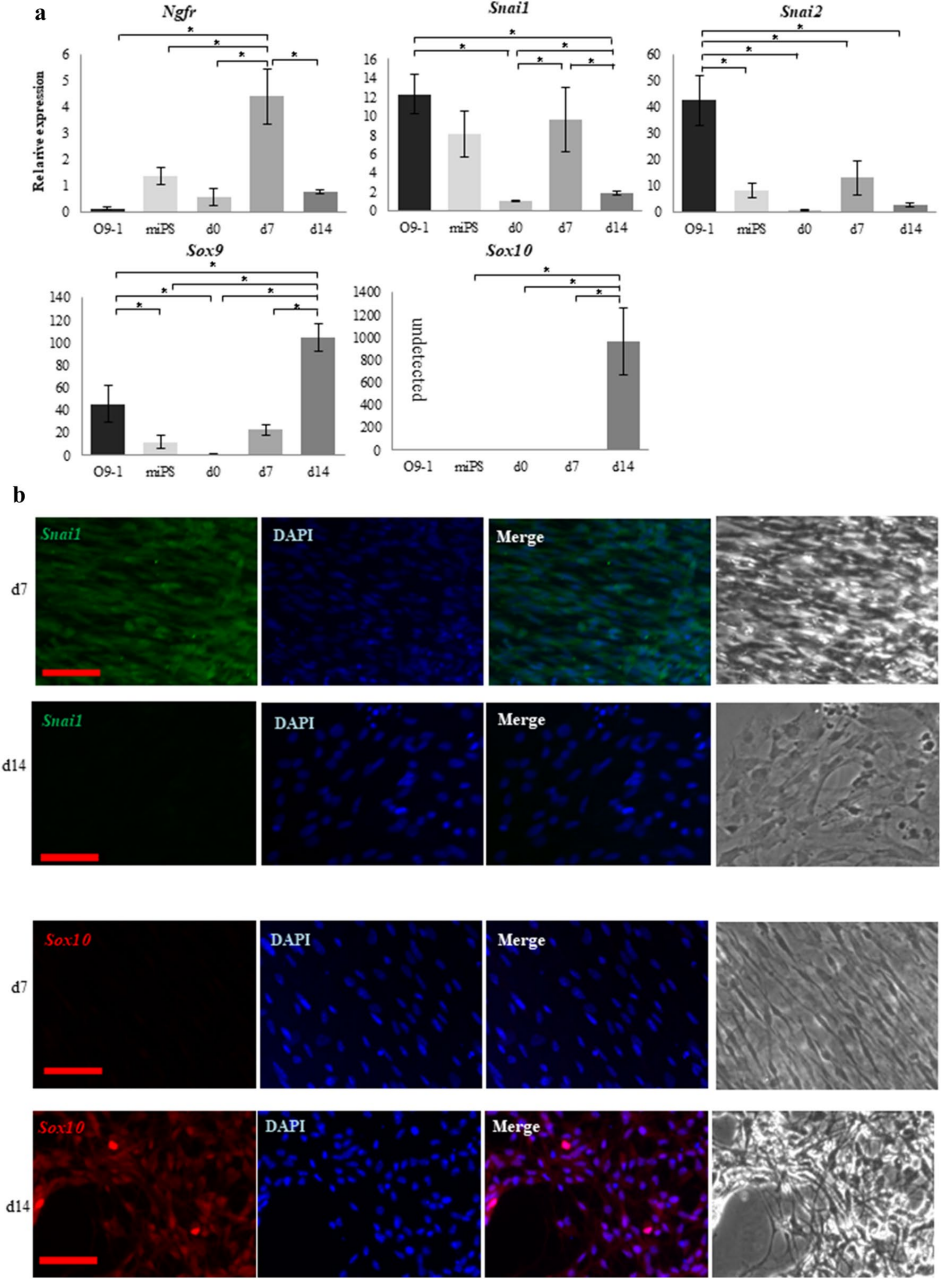
Comparison between O9-1 cells and cranial neural crest cells (cNCCs) derived from mouse-induced pluripotent stem (miPS) cells using quantitative reverse transcription polymerase chain reaction (qRT-PCR) and immunostaining. **a** Expression of the premigratory neural crest (NC) markers *Ngfr*, *Snai1*, and *Snai2* and the migratory NC and cNC markers *Sox9* and *Sox10*. Expressions of the premigratory NC markers increased in day 7 (d7) cells, whereas those of the migratory markers increased in d14 cells. *Sox10* was not detected in O9-1 cells. Each experiment was performed in triplicate, with values representing mean ± SD. Groups were compared using ANOVA, followed by the Bonferroni test: *p < 0.05. **b** Immunostaining of d7 and d14 cells. *Sox10* was more highly expressed in the d14 cells, whereas *Snai1* was more highly expressed in the d7 cells. Scale bar 50 μm

Expressions of the NC markers *Ngfr*, *Snai1*, *Snai2*, *Sox9*, and *Sox10* were examined by qRT-PCR in cNCCs derived from miPS cells as well as in O9-1 cells as a control. Expression of all genes except *Ngfr* and *Sox10* was detected in O9-1 cells [24]. In contrast, expressions of all genes were detected in cNCCs, with the premigratory NC markers *Ngfr*, *Snai1*, and *Snai2* exhibiting the highest expression levels in d7 cells and the migratory and cranial NC markers *Sox9* and *Sox10* exhibiting the highest expression levels in d14 cells (Fig. 2a). The strongest immunofluorescent staining was detected for *Snai1* and *Sox10* in d7 and d14 cells, respectively (Fig. 2b).

### NC specifier transcription factors

**Fig. 3:**
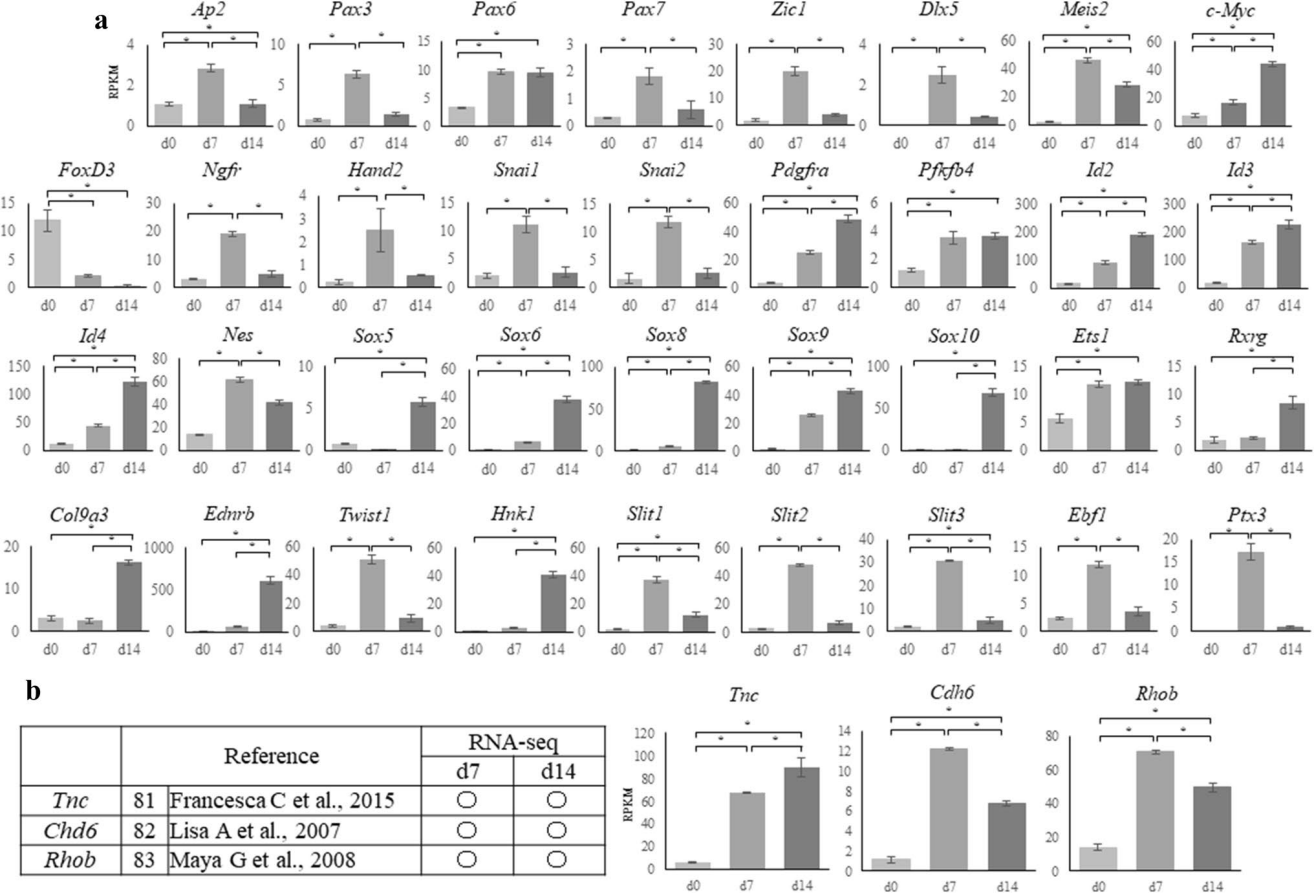
RNA sequencing results for cranial neural crest cells (cNCCs) differentiated from mouse-induced pluripotent stem (miPS) cells. **a** Expression of each of the genes listed in Table 2 at day 0 (d0), d7, and d14 after induction. Sex-determining region Y (SRY)-related high mobility group (HMG) box genes showed the highest upregulation in d14 cells. The vertical axis reveals reads per kilobase of exon per million mapped reads (RPKM), and the horizontal axis indicates time. Each experiment was performed in triplicate, with values representing mean ± SD. Groups were compared using ANOVA, followed by the Bonferroni test: *p < 0.05. **b** Expression of genes that have not been examined during the neural crest stages in vivo. *Tnc* showed the highest upregulation in d14 cells, whereas *Cha6* and *Rhob* were upregulated in day 7 (d7) cells. The vertical axis indicates reads per kilobase of exon per million mapped reads (RPKM), and the horizontal axis indicates time. Open circles indicate genes upregulated in d7 or d14 compared with d0 [log fold change (FC) > 1, *p* < 0.01, false discovery rate (FDR) < 0.05)]. Each experiment was performed in triplicate, with values representing mean ± SD. Groups were compared using ANOVA, followed by the Bonferroni test: *p < 0.05

We conducted a literature search of NC specifier transcription factors identified in vivo [16, 25–80] (Tables 2, 3) and compared these reports with our RNA-seq results. The relative expressions of genes that underwent a significant change in expression are presented in Fig. 3a.

**Table 2:**
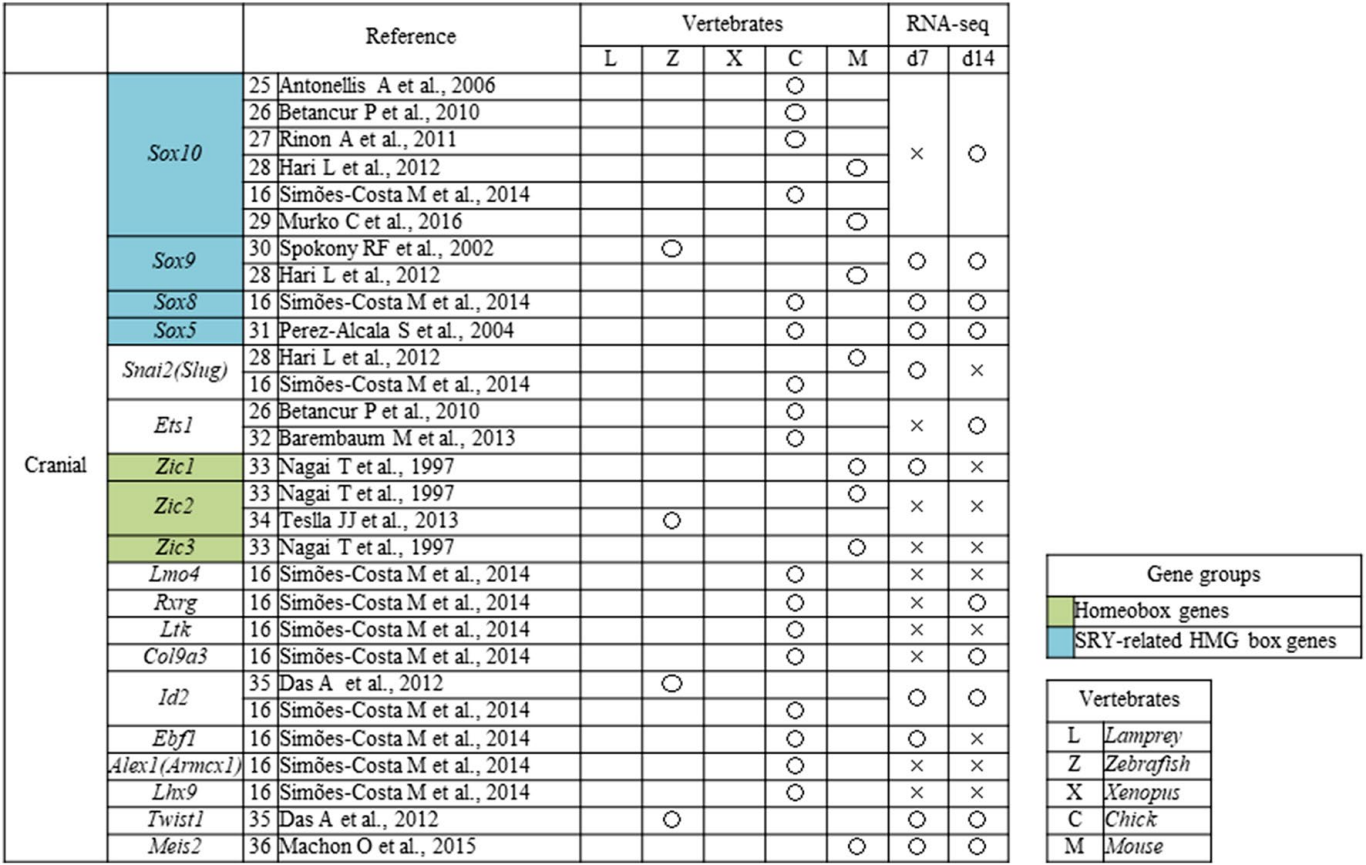
Neural crest (NC) genes that have previously been examined in vivo

Open circles indicate genes that were upregulated on day 7 (d7) or d14 compared with d0 [log fold change (FC) > 1, *p* < 0.01, false discovery rate (FDR) < 0.05), whereas crosses indicate genes that were not upregulated

**Table 3:**
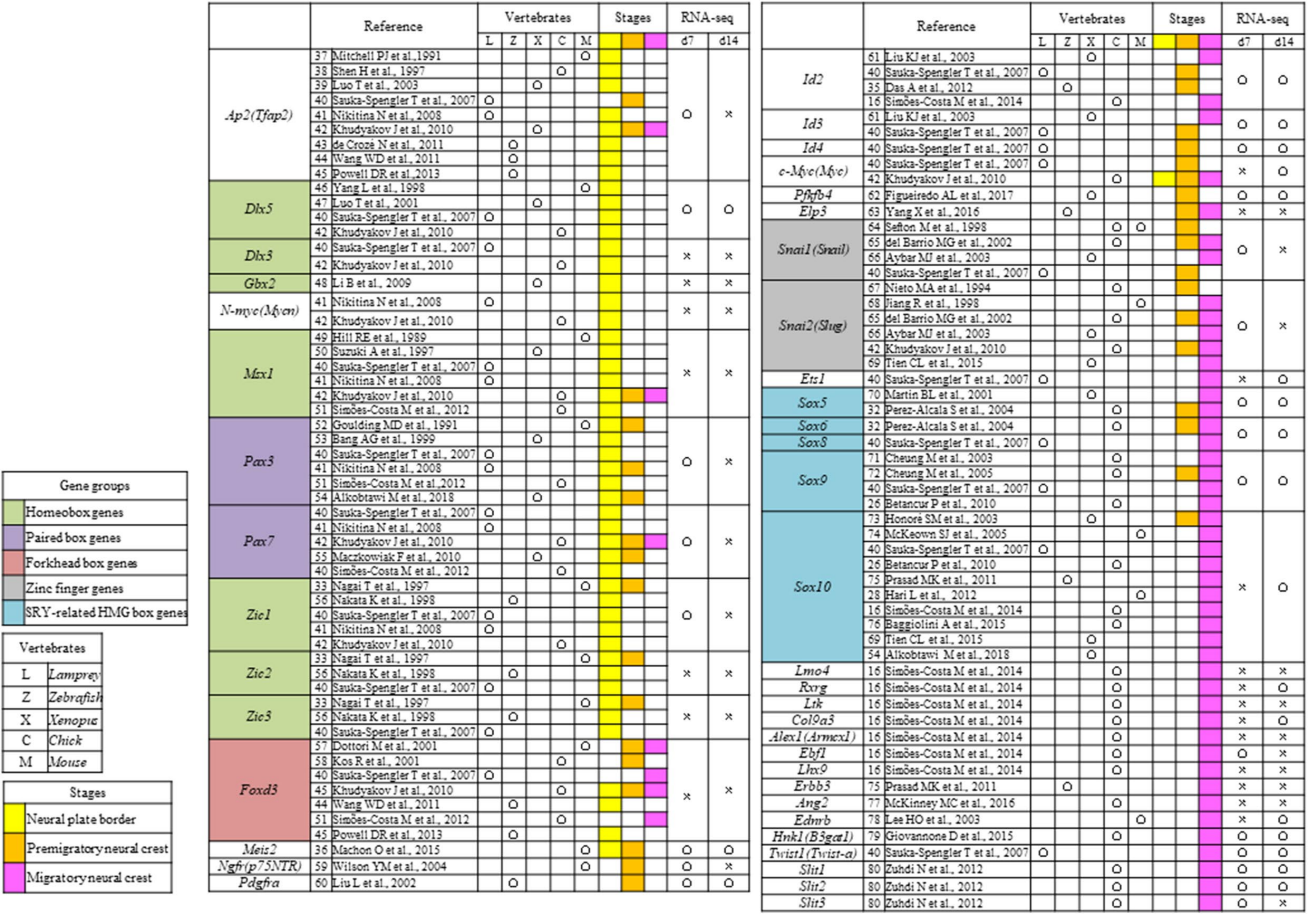
Neural crest (NC) transcription factors that have previously been examined in vivo

Open circles indicate genes that were upregulated on day 7 (d7) or d14 compared with d0 [log fold change (FC) > 1, *p* < 0.01, false discovery rate (FDR) < 0.05), whereas crosses indicate genes that were not upregulated

The *transcription factor AP*-*2 alpha* (*Ap2*) along with paired box 3 (*Pax3*) and zinc finger protein of the cerebellum 1 (*Zic1*), both of which are regulated by *Ap2*, were the most highly expressed genes in d7 cells (Fig. 3a). *Pax6*, which has been reported in human ES and iPS-derived NC cells (Tables 2, 3), was detected in both d7 and d14 cells, whereas *Pax7*, which has not previously been reported in the mouse NC, was detected in the d7 cells (Fig. 3a). In contrast, the homeobox genes *gastrulation brain homeobox 2 (Gbx2),* msh homeobox 1 (*Msx1*), *distal*-*less homeobox 3 (Dlx3), Zic2*, and *Zic3* were not detected in d7 or d14 cells, and the homeobox genes *Zic1* and *Dlx5* were only expressed in the d7 cells, despite these having been reported in the NC of a range of species (Table 2); however, *Meis homeobox 2 (Meis2)* was expressed in both d7 and d14 cells.

The MYCN proto-oncogenes, bHLH transcription factor (*N*-*myc*) and *c*-*Myc*, have been reported in NCCs (Table 3); however, *c*-*Myc* expression was detected in d7 and d14 cells (Fig. 3a), while *N*-*myc* was not. Furthermore, there was a gradual and substantial downregulation of the winged-helix transcription factor Forkhead box D3 (*FoxD3*) (Fig. 3a), which is an important factor for maintaining the pluripotency of ES cells and a key NC specifier that has been implicated in multiple stages of NC development and NCC migration in embryos of various species (Tables 2, 4).

**Table 4:**
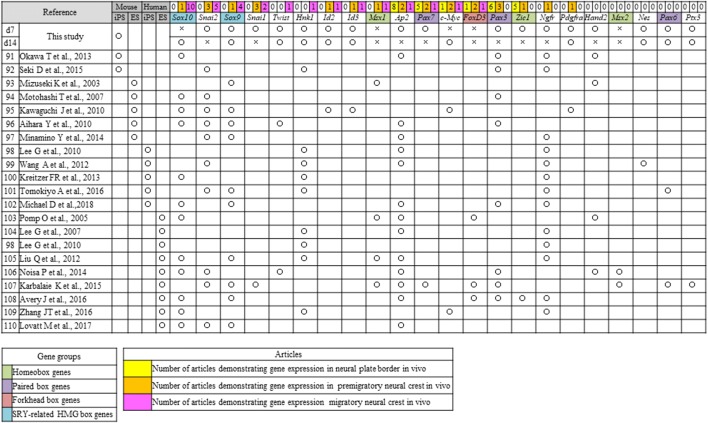
Neural crest (NC) transcription factors that have previously been examined in vitro

Open circles indicate genes that were upregulated on day 7 (d7) or d14 compared with d0 [log fold change (FC) > 1, *p* < 0.01, false discovery rate (FDR) < 0.05), whereas crosses indicate genes that were not upregulated

The premigratory NC markers *Ngfr*, heart and neural crest derivatives expressed 2 (*Hand2*), *Snai1*, and *Snai2* were only detected in the d7 cells; however, other premigratory NC markers, such as the platelet derived growth factor receptor, alpha polypeptide (*Pdgfra*); 6-phosphofructo-2-kinase/fructose-2,6-biphosphatase 4 (*Pfkfb4*); inhibitor of DNA binding 2 (*Id2*), *Id3*, and *Id4*; and nestin (*Nes)* were detected in both d7 and d14 cells (Fig. 3a).

Expression of migratory NC markers such as *Sox5*, -*6*, -*8*, -*9*, and -*10*, which encode members of the sex-determining region Y (SRY)-related high mobility group (HMG)-box family of transcription factors and are crucial in several aspects of NCCs, were detected in d7 or d14 cells. *Sox10*, a known marker for migratory cNCCs in various species (Table 2), was only detected in d14 cells similar to the other migratory NC markers. Twist family bHLH transcription factor 1 (*Twist1*), which is activated via various signal transduction pathways and is crucial for E-cadherin downregulation, as well as beta-1,3-glucuronyltransferase 1 (*B3gat1*/*Hnk1*), which plays a role in the formation of CD57 epitope, was detected in both d7 and d14 cells. In contrast, the expression of the trunk NC markers lit guidance ligand 1/2 (*Slit1/2*), which plays an important role in trunk NC cell migration toward ventral sites, was upregulated only in d7 cells (Fig. 3a).

Finally, expressions of tenascin C (*Tnc*), cadherin-6 (*Cdh6*), and ras homolog family member B (*Rhob*), all of which are related to cell adhesion and motility [81–85], were significantly increased in both d7 and d14 cells (Fig. 3b).

### Metzincin superfamily zinc proteinase and protocadherin superfamily

**Fig. 4:**
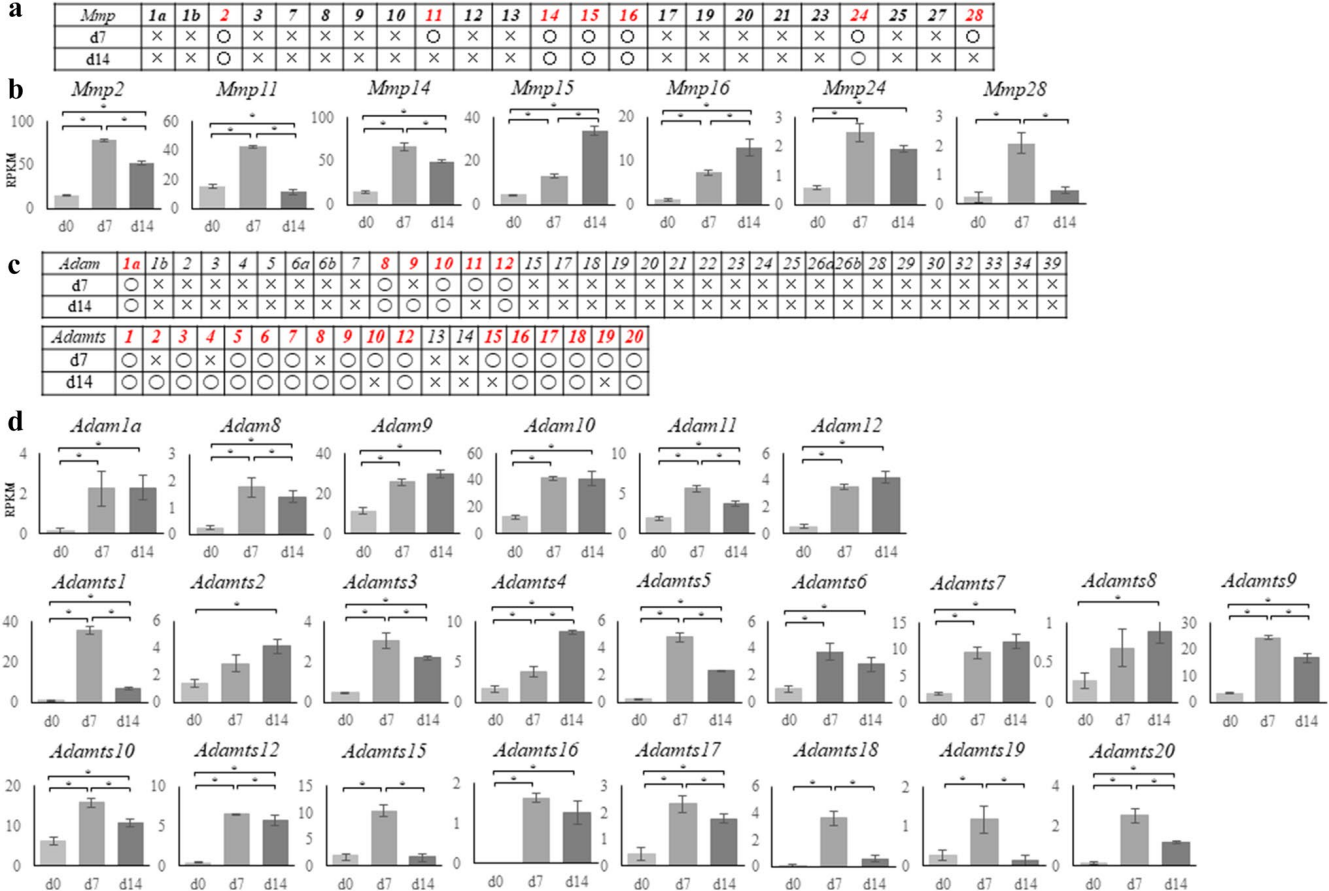
RNA sequencing results for the matrix metalloproteinase (*Mmp*), a disintegrin and metalloproteinase (*Adam*), and a disintegrin and metalloproteinase with thrombospondin motifs (*Adamts*) gene families. **a** Expressions of *Mmp* family genes in mouse. Round marks alongside day 7 (d7) or d14 cells indicate that the genes were upregulated compared with d0 [log fold change (logFC) > 1, *p* < 0.01, false discovery rate (FDR) < 0.05], whereas cross marks indicate lack of upregulation. **b** Graphical representation of the upregulation of *Mmp2*, -*11*, -*14*, -*15*, -*16*, -*24*, and -*28* in d7 or d14 cells. *Mmp15* and -*16* showed the highest upregulation in d14 cells. The vertical axis indicates reads per kilobase of exon per million mapped reads (RPKM), and the horizontal axis indicates time. Each experiment was performed in triplicate, with values representing mean ± SD. Groups were compared using ANOVA, followed by the Bonferroni test: *p < 0.05. **c** Expressions of *Adam* and *Adamts* genes in mouse. Round marks alongside d7 or d14 cells indicate that the genes were upregulated compared with d0 (logFC > 1, *p *< 0.01, FDR < 0.05), whereas cross marks indicate lack of upregulation. **d** Graphical representation of the upregulation of *Adam1a* and *8*–*12*, and *Adamts1*–*10*, -*12*, and *15*–*20* in the d7 or d14 cells. *Adam2*, -*4*, -*7*, and -*8*, and *Adamts 9* and -*12* showed the highest upregulation in d14 cells. The vertical axis indicates reads per kilobase of exon per million mapped reads (RPKM), and the horizontal axis indicates time. Each experiment was performed in triplicate, with values representing mean ± SD. Groups were compared using ANOVA, followed by the Bonferroni test: *p < 0.05

Members of the metzincin superfamily are proteinases that carry a zinc ion at their active site. This family includes the matrix metalloproteinases (Mmps), Adam, and Adamts, all of which have gained attention as factors involved in cancer cell invasion and migration. *Mmp2*, -*11*, -*14*, -*15*, -*16*, -*24*, and -*28* were significantly upregulated in cNCCs (Fig. 4a), all of which except *Mmp24* are membrane-bound. *Mmp11* and -*28* were only expressed in d7 cells, whereas all other Mmps were detected in both d7 and d14 cells (Fig. 4a, b).

Only *Adam1a*, -*8*, -*10*, and -*12* were upregulated in both d7 and d14 cells (Fig. 4c, d); this is contrary to reports that the members of this family are important in NC migration and that *Adam*-*10*, -*12*, -*15*, -*19*, and -*33* are expressed in the mouse NC [86]. Moreover, various *Adamts* family genes, which are important for connective tissue organization and cell migration, were upregulated in either d7 or d14 cells (Fig. 4c, d). In particular, *Adamts1* expression was markedly increased, whereas *Adamts2* and -*8* expressions, which are presumably important in cancer cell invasion [87–89], increased in the later stages of differentiation.

Most *Pcdh* genes, which are involved in cell adhesion [90], were upregulated in d7 and d14 cells (Table 5); however, *Pcdha2*, -*5*, -*7*, -*11*, and -*12*; *Pcdhac1*; and *Pcdhgc5* were only upregulated in d14 cells.

**Table 5:**
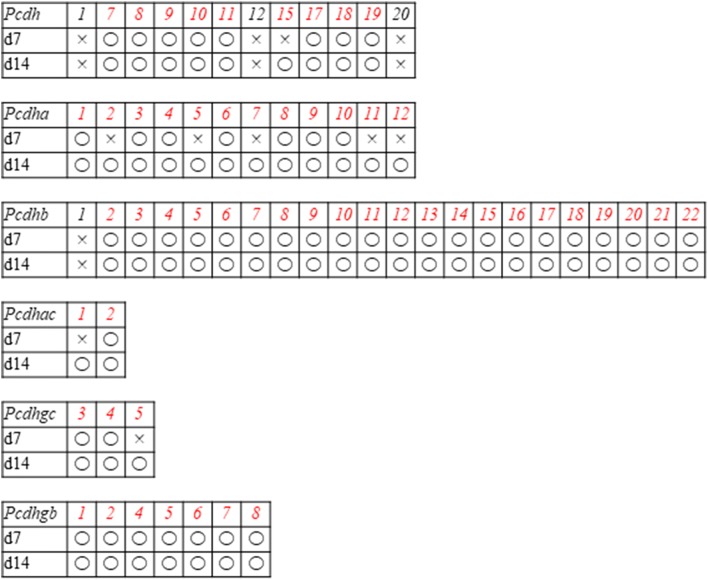
Expression of the protocadherin superfamily based on RNA sequencing data

Open circles indicate genes that were upregulated on day 7 (d7) or d14 compared with d0 [log fold change (FC) > 1, *p* < 0.01, false discovery rate (FDR) < 0.05), whereas crosses indicate genes that were not upregulated

## Discussion

In the present study, we derived cells from miPS which are closely migratory cNCCs genes. Previously, NCCs have been derived from ES or iPS cells using various approaches [91–110], and the protocol used in the present study was based on the methods outlined by Bajpai et al. [23]; however, few studies have investigated changes in the properties of cNCCs at different time points after induction.

In the present study, d7 and d14 cells expressed typical NC markers, such as *Ngfr*, *Snai1*, and *Snai2*. In contrast, O9-1 cells (controls) did not express *Ngfr*, suggesting that cNCCs derived from miPS cells are better than O9-1 cells for evaluating cNCC characteristics [24]. Moreover, unlike O9-1 cells, d14 cells expressed markedly high levels of *Sox10*, which is considered a reliable marker for migratory cNCCs. Since cNCCs are involved in craniofacial tissue organization, several reports are available on their gene expression profiles; however, these reports show varying results with species and protocols. Moreover, cNCCs rapidly differentiate in the embryo [14]; thus, it is considerably difficult to synchronize the timing of isolation to a particular point during their development. Furthermore, migratory cNCCs intermingle with other cell types in the embryo, further complicating the isolation and characterization of a pure cell population. Consequently, there have been few reports on cNCC markers [16, 25–36]. Simões-Costa et al. [16] successfully isolated *Sox10*-positive cNCCs from chicken embryos and analyzed their gene profiles. Similarly, we detected *Sox10* expression in d14 cNCCs. Reportedly, there are multiple NCC populations [11], and iPS cells can differentiate into numerous different NCC populations in the same culture. Therefore, this diversity in populations may explain the discrepancies in results; however, under the conditions used in the present study, *c*-*Myc*; *Ets1*; *Sox10*; *Adamts2*; *Adamts8*; *Pcdha2*, -*5*, -*7*, -*11*, and -*12*; *Pcdhac1*, and *Pcdhgc3* may represent useful markers for migratory cNCCs. Furthermore, our results indicated that d7 cells were in the premigratory stage despite expressing numerous NC markers. Therefore, cNCCs derived from miPS cells required > 14 days to become migratory in vitro, and this duration is considerably longer than that observed in the mouse embryos in vivo under the same conditions [111].

The use of RNA-seq facilitates the normalization of expression levels of different genes, allowing comparisons between samples. In our triplicate experiments, none of the induced cNCCs expressed several homeobox genes considered to be expressed in the early stages of cNCC differentiation. In particular, we did not observe *FoxD3* expression in either d7 or d14 cells, although it has been recognized as one of the key transcription factors in cNCCs [112]. These contradictory results suggest that cNCCs derived from miPS cells express distinct gene regulatory networks. *FoxD3*, a pluripotent stem cell marker gene that plays an important role in maintaining pluripotency, is expressed at different time points in different cells, but its expression decreases in a time-dependent manner [41], indicating that *FoxD3* may not be a key regulator in iPS-derived cNCCs. However, we speculate that iPS cells express sufficient levels of *FoxD3* to differentiate into cNCCs.

Protocadherins belong to the cadherin superfamily and are involved in intercellular interactions [90], whereas metzincins are key proteinases that facilitate cell migration [42]. Unfortunately, the abundances of members of these families hindered their analysis; however, because RNA-seq enabled us to comprehensively evaluate the gene expression profiles, we were able to focus on expressions of all procadherin and metazicin family members. As expected, we observed that several *Adam* and *Adamts* genes were upregulated, with most of the *Admats* genes showing significantly increased expression. The *Adam* genes with increased expression in cNCCs were membrane-bound, whereas *Adamts* genes which secreted proteinases, indicating that the expression of various *Adamts* may allow the matrix to be digested more efficiently and that each proteinase may be capable of digesting a different type of extracellular matrix protein [42]. Therefore, the secretion of various Adamts and Pcdh proteins may play a crucial role in cNCC migration.

## Conclusion

In summary, cNCCs derived from miPS exhibited RNA expression profiles that partly overlap with previously reported profiles. These cells may be useful for the regeneration of tissue formed by NCCs (osteoblast, melanocyte, and glial cells). We observed that although the resulting cNCCs exhibited several NC specifiers, they lacked some of the specifiers, indicating that a distinct molecular network may regulate gene expression in miPS-derived cNCCs. Moreover, our results indicated that *c*-*Myc*; *Ets1*; *Sox10*; *Adamts2* and -*8*; *Pcdha2*, -*5*,-*7*, -*11*, and -*12*; *Pcdhac1*; and *Pcdhgc3* may represent appropriate markers for migratory miPS-derived cNCCs. Finally, cNCCs expressed a wide spectrum of genes encoding Adamts family enzymes that may be crucial for their migration.

## Electronic supplementary material

Below is the link to the electronic supplementary material.

Comparison between the positive control and the negative control using immunostaining and quantitative reverse transcription polymerase chain reaction (qRT-PCR). (a) Immunostaining of positive control and negative control. The positive control of Snai1 was O9-1 cells and the positive control of Sox10 was DP cells. The negative control of Snai1 and Sox10 was SNL cells. Scale bar = 50 μm. (b) Expressions of *Snai1* and *Sox10* increased in the positive control. Each experiment was performed in triplicate, with values representing mean ± SD. Groups were compared using ANOVA, followed by the Student’s *t* test: *p < 0.05. (DOCX 391 kb)
