# Supplementary figures and images for: Stage-dependent differential gene expression profiles of cranial neural crest-like cells derived from mouse-induced pluripotent stem cells
Source: Med Mol Morphol. 2019 Jul 11;53(1):28–41. doi: 10.1007/s00795-019-00229-2 (PMC7033077; doi:10.1007/s00795-019-00229-2)

Fig. S1


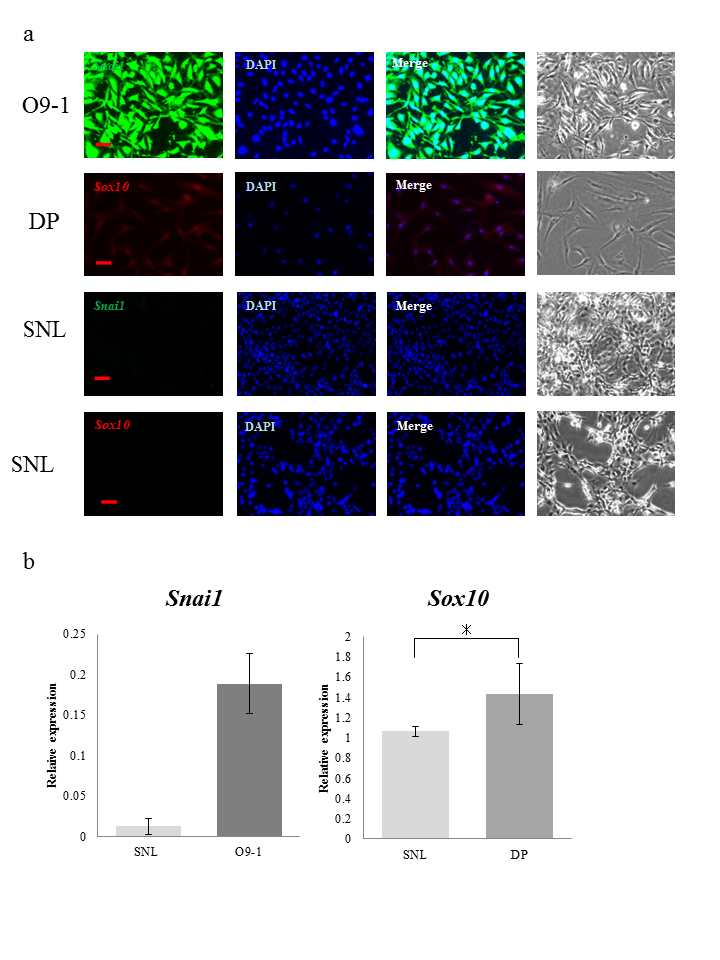

Supplement: Supplementary file 1 — Comparison between the positive control and the negative control using immunostaining and quantitative reverse transcription polymerase chain reaction (qRT-PCR). (a) Immunostaining of positive control and negative control. The positive control of Snai1 was O9-1 cells and the positive control of Sox10 was DP cells. The negative control of Snai1 and Sox10 was SNL cells. Scale bar = 50 μm. (b) Expressions of Snai1 and Sox10 increased in the positive control. Each experiment was performed in triplicate, with values representing mean ± SD. Groups were compared using ANOVA, followed by the Student’s t test: *p < 0.05. (DOCX 391 kb) [file 795_2019_229_MOESM1_ESM.docx]
